# Development of a Maternal, Newborn and Child mHealth Intervention in Thai Nguyen Province, Vietnam: Protocol for the mMom Project

**DOI:** 10.2196/resprot.7912

**Published:** 2018-01-11

**Authors:** Bronwyn McBride, Liem Thanh Nguyen, David Wiljer, Nguyen C Vu, Cuong K Nguyen, John O'Neil

**Affiliations:** ^1^ Faculty of Health Sciences Simon Fraser University Burnaby, BC Canada; ^2^ Gender and Sexual Health Initiative British Columbia Centre for Excellence in HIV/AIDS Vancouver, BC Canada; ^3^ Institute of Population, Health and Development Hanoi Viet Nam; ^4^ Nossal Institute for Global Health University of Melbourne Melbourne Australia; ^5^ Education Technology Innovation University Health Network Toronto, ON Canada; ^6^ Institute for Health Policy Management and Evaluation Department of Psychiatry University of Toronto Toronto, ON Canada; ^7^ Vietnam eHealth Medical Investment and Communication Hanoi Viet Nam

**Keywords:** mobile health, Vietnam, maternal health, reproductive health, health equity

## Abstract

**Background:**

Ethnic minority women (EMW) living in mountainous areas of northern Vietnam have disproportionately high infant and maternal mortality rates as a result of low maternal health knowledge, poverty, and remoteness from low-capacity health centers.

**Objective:**

The objective of this study was to describe the protocol for the development and evaluation of the mMom intervention, which is an integrated mobile health (mHealth) system designed to improve maternal and infant health knowledge, and behavior among women in remote areas of Thai Nguyen, Vietnam.

**Methods:**

This project featured the following four phases: (1) development of an mHealth platform integrated into the existing health management information system in partnership with the provincial health department; (2) ethnographic fieldwork and intervention content development; (3) intervention piloting and implementation; and (4) evaluation of the intervention’s impact on participants’ maternal health knowledge, behavior, and interactions with the health system.

**Results:**

The mMom project development process resulted in the following: (1) the successful development of the mMom system, including the mHealth platform hardware and integration, the intervention plan and content, and the monitoring and evaluation framework; (2) the piloting and implementation of the intervention as planned; and (3) the implementation of the monitoring and evaluation framework components.

**Conclusions:**

This protocol outlines the development of the mMom intervention and describes critical next steps in understanding the impact of the intervention on participants and the wider health system in Thai Nguyen province, Vietnam.

## Introduction

### Background

Vietnam has made remarkable progress in improving maternal, newborn, and child health (MNCH), but ethnic minority women (EMW) living in mountainous and remote areas still lag far behind. Although Vietnam’s overall under-five mortality rate declined from 50.8 per thousand live births in 1990 to 21.7 per thousand in 2015 [1] and the maternal mortality rate dropped from 139 per hundred thousand live births in 1990 to 54 per hundred thousand in 2014 [2], disaggregated data from the last three censuses of Vietnam show that ethnic minority people have higher total fertility rates, higher infant and child mortality rates, lower life expectancies, and are less likely to attend antenatal or postnatal care or to deliver in a health facility than Kinh people (the ethnic majority group) [3,4]. In 2009, the national total fertility rate was 2 children per woman but 5 for H’Mong minority women. H’Mong women also experience the highest infant mortality rate in Vietnam at 46 per thousand live births, whereas the infant mortality rate in Kinh women is 12 per thousand [5]. Undernutrition and stunting rates of children in mountainous areas, which have high concentrations of ethnic minority people, are 3 times higher than those in the wealthier lowland provinces, and maternal mortality rates are also significantly higher among EMW compared with Kinh women, at 316 and 81 per hundred thousand births, respectively [6]. In 2011, 55% of EMW nationwide reported that they had given birth in a health facility, in contrast to over 95% of Kinh women [7]. Although Kinh people have vaccination rates of 73%, the average rate across ethnic minority groups is 38% [3].

Health outcome disparities among ethnic minorities in northern Vietnam are profound, primarily because of sociostructural factors including poor education, low economic and social status, and rural or remote residence [4-6,8-10]. Determinants influencing the substantially poorer MNCH and higher infant mortality rates among EMW and their newborns are limited access to information, low reproductive health knowledge, poor MNCH behaviors, poor access to and uptake of perinatal and postnatal care services, and language barriers [8-11]. In addition, health facilities are unevenly distributed throughout Vietnam, with fewer and lower capacity facilities and staff in remote regions [10,11]. Specific contributing factors include relatively low uptake of tetanus vaccinations among EMW during pregnancy and low uptake of modern contraceptives [7]. In Thai Nguyen province, where ethnic minorities constitute 27% of a population of 1.3 million [12] and where the majority live in mountainous and remote areas, the situation is particularly acute.

### Current Approaches to Global Mobile Health

Over the past decade, mobile health (mHealth) initiatives have burgeoned in both low- and high-income contexts, supported by the high penetration of inexpensive mobile phone services. mHealth has been widely applied to address the stark global inequities in maternal and infant mortality in low- and middle-income countries, as interconnected economic, political and sociocultural factors have impeded progress on MNCH indicators and mHealth may hold the potential to mitigate some of these barriers.

With regard to mHealth acceptability, several recent studies in diverse regions have identified receptive attitudes from target communities toward mHealth projects, ranging from 70% to 90% in different interventions [13-15]. To date, most mHealth initiatives described in the literature are associated with positive health outcomes. A 2014 systematic review of mHealth interventions in low- and middle-income countries found that mHealth has been effective in improving treatment adherence, appointment compliance, data gathering, and developing support networks for health workers [16]. In addition, reviews of recent initiatives show that it has become a powerful tool with the potential to reduce disparities, improve the quality of services delivered by health care workers, and improve access to health services leading to improvement of MNCH in developing countries [17-22]. A review of current mHealth initiatives suggests that mHealth projects are generating benefits in the areas of surveillance of MNCH in India, quality MNCH services in Ghana, MNCH information and education in Tanzania [23], family planning information provision in Kenya and Tanzania [24], and quality health services in Sierra Leone [25]. Among various initiatives using short message service (SMS) for MNCH, the Mobile Alliance for Maternal Action (MAMA), initiated in May 2011, provides critical stage-based information to new and expectant mothers in Bangladesh, India, and South Africa via mobile phones [20]. MAMA has a library providing free adaptable mHealth reminders for programs that are using mobile phones to inform and empower new and expectant mothers.

Available literature indicates high acceptability of mHealth among health staff and that mHealth projects can positively impact community health work and systems. mHealth interventions have been found to increase staff efficiency and improve data entry and monitoring [26-28], though sufficient staff training has been a challenge in some interventions [29]. An SMS intervention in Malawi reduced facility admissions for minor health issues such as fever, thereby decreasing health staff’s workload through supporting participants in treating minor conditions at home [26]. A study of health worker perspectives in rural Rwanda found that the use of the RapidSMS mHealth technology to reduce maternal and newborn deaths improved health workers’ interactions with mothers and that health workers’ knowledge increased, with community health workers reporting improved mobile phone usage skills and increased knowledge of MNCH [29].

Although there has been an increased application of mHealth to support MNCH, evidence of the outcomes of SMS-based interventions for MNCH is still limited [30]. Tamrat and Kachnowski argue that the use of mHealth in MNCH is underdeveloped, and their review suggests that studies of ongoing programs are needed [31]. Furthermore, in comparison with the wider adoption of mHealth in emergency care, point of care support, health promotion, and data collection, there are few studies that examine its use for improving prenatal or neonatal access to health care [31]. Studies in Indonesia and Ghana demonstrated that midwives with low literacy levels could use text messaging effectively to convey information [32,33], and a study in Tanzania found that pregnant women linked to health units by mobile phone had increased contact with midwives and were more likely to have a skilled attendant at delivery [34].

Vietnam’s mobile phone network and market have undergone swift development as the country has experienced rapid economic growth since the late 1980s. Vietnam is categorized as a low-income country, but the number of mobile subscriptions over its population is higher than that of many developed countries. In 2015, there were 130 mobile cellular subscriptions for every 100 people in Vietnam [35] and a total of 122 million mobile subscribers over a total population of 93.4 million [36]. SMS has been used widely for advertisement and commercial services but not for social services, and the application of mHealth in Vietnam is still limited. Mobile service coverage is wide and costs are low (ie, 500 Vietnam dong or US $2.5 cents per SMS sent, and free receipt of SMS) and as such, mHealth holds great potential for affordable provision of social services to hard-to-reach populations, such as ethnic minority groups and people living in remote regions. However, to date, mHealth appears to be underutilized in Vietnam in contrast to other regions in Asia. To our knowledge, mHealth has only been applied to provide health information to internal migrants in Vietnam [37] and has not yet been used to support maternal health with the exception of the mMom project.

Furthermore, although literature on the development of mHealth interventions is growing, certain areas remain understudied. A gap in current literature is the relative absence of studies examining policy and management frameworks to support adoption of mHealth services, with a specific gap related to governance and partnerships with regional and national partners. Issues such as the coordination for mHealth projects among different government bureaus, and protocols for assuring quality of information, require further investigation. This study aimed to address this noted gap through creating multilevel partnerships between commune-, district-, and provincial-level health authorities and documenting the challenges of an integrated, intergovernmental, and interdepartmental project to contribute lessons currently lacking in the literature.

The objective of this paper is to describe the protocol for the development and evaluation of the mMom intervention, which is an integrated mHealth system designed to improve maternal and infant health knowledge and behavior among women in a remote area of Thai Nguyen province in mountainous northern Vietnam.

## Methods

### Development of an mHealth Platform Integrated Into the Existing Health Management Information System in Partnership With the Provincial Health Department

The Government of Vietnam has demonstrated increasing interest in health system strengthening and mHealth over the past decade, creating a favorable policy environment and suitable context for partnerships toward greater integration of mHealth. In 2009, the Ministry of Health established a Central Health Information and Technology Institute, and telemedicine and Web-based electronic medical records systems were subsequently established to strengthen health management information systems (HMISs). In 2012, the Ministry of Health introduced a new set of administrative directives on medical and health information technology (IT), and the Thai Nguyen Provincial Health Department (TNHD) received funds from Atlantic Philanthropy to computerize its HMIS, supported by the Institute of Population Health and Development (PHAD). In Thai Nguyen province, all hospitals and 181 communes in 9 districts are fully integrated into an electronic record management system, and 100% of commune health centers are connected by high speed cables to district and provincial health centers.

Due to the central and provincial government’s commitments to use IT to improve health, the mMom project fit well within national and local priorities. The mMom software and its operation was developed and managed by Vietnam electronic health (eHealth) Medical Investment and Communication. The project built a new mHealth component, aimed at health communication, as part of the existing HMIS for maternal and child health. However, this component can also function independently and can be applied to other modules of the HMIS. The mMom database was created as an integrated component of the HMIS through using each patient’s unique identity code. The platform aimed to coordinate and add support to commune health workers’ existing tasks in monitoring pregnancy and new motherhood in community women. In addition, the project’s integration into the HMIS aimed to improve data collection and monitoring, with the goal of supporting future MNCH programming evaluation and improvements due to strengthened data collection systems.

### Ethnographic Fieldwork and Intervention Content Development

Ethnographic fieldwork was conducted at the mMom project outset to identify gaps in current MNCH approaches in the intervention site (Dinh Hoa district, Thai Nguyen, Vietnam) and to inform the mHealth intervention. Fieldwork methodology included focus group discussions (bringing people together to explore their attitudes and experiences on a topic of interest in a group setting); in-depth interviews (guided conversations between a researcher and an individual, which collect specific information on their perceptions and experiences) with the target populations of currently pregnant women, new mothers, their families, and health workers; and document review. Focus groups and interviews were selected as the methods most suited to achieving the fieldwork goals of assessing the extent of mobile phone use in the district, literacy barriers, social and cultural issues affecting phone ownership and use, health service utilization, capacity and response of health workers, organizational factors that may impact mHealth promotion, and technical constraints such as mobile signal coverage.

The intervention was developed based on several well-known conceptual frameworks for mHealth and maternal health. These included the following: (1) the World Health Organization (WHO) framework and standards for country health information systems, which emphasizes reliable and timely health information as an essential foundation of effective public health systems and provides guidelines for strengthening the key components of health information systems [38]; (2) the Philbrick strategic framework on mHealth for MNCH, which proposes 3 strategic objectives that lead to improving MNCH in low- and middle-income countries and 5 operational priorities for public health professionals and policy makers to conceptualize how mobile technology can be harnessed to improve MNCH and to increase its effective use [17]; (3) the Mobile Alliance for Maternal Health Global Monitoring and Evaluation Framework, a tool for planning, management, monitoring and evaluation (M&E) of mHealth projects to ensure that programs meet the needs of the target population [20]; and (4) the WHO mHealth framework for assessing the impact of MNCH mHealth interventions on health systems, which describes the value of mHealth solutions in strengthening health systems across reproductive, maternal, neonatal, and child health areas and combines metrics on health system functions and needs and matches them to appropriate mHealth strategies [39]. These frameworks were used as reference or for guidance during development of the project intervention, logical framework, and indicators under a results-based management accountability framework and will be used for project impact assessment. The mMom project was developed by a team of local and international experts and was funded by the International Development Research Centre (IDRC)—a Canadian funding organization that supports research on capacity development in low- and middle-income countries. The project aimed to build on prior successful collaborative work on HMIS among the PHAD, Population Council, and TNHD.

The intervention consists of 2 SMS message programs designed to support women’s health during pregnancy and new motherhood. The mMom SMS messages were developed using MAMA [20] materials as a primary reference. From the MAMA templates, messages that were applicable to the context of northern Vietnam were selected and then further adapted for local acceptability among the project’s target population. Additional messages based on the Vietnam Ministry of Health guidelines on MNCH were added. The TNHD and external experts provided feedback on the message content, and the messages were piloted with members of the target population in nonstudy sites before being finalized for the intervention. The first SMS program was focused on prenatal care and offered actionable tips on how to remain healthy throughout pregnancy and promote healthy fetal development. Topics included what to expect during each stage of pregnancy, danger signs, antenatal care visits, nutrition, supplements, and immunizations. The program featured a total of 75 SMS messages, which were delivered 2 to 3 times per week from the 5th to 42nd week of pregnancy. The second program was initiated once the infant was born and provided information on women’s postpartum care and infant development. Topics included breastfeeding, danger signs, immunization for baby, and contraception. The program included 71 messages, which were delivered 1 to 2 times per week for the first year of the baby’s life. The mMom messages were brief, written in accessible language, and sent automatically by the database to all participants.

Each SMS program was designed to include the following 4 types of messages: informational and educational, reminder, interactive, and scanning. Informational, educational and reminder messages provided one-way information and reminded women to take critical actions, such as getting a tetanus immunization. Interactive or three-way messages requested women to respond to monitoring questions, such as whether the participant has recently visited the health center. If women do not respond, or if their responses suggest a risk, the database automatically informs that individual participant’s health worker so that they may reach out to the woman. Within each SMS program, approximately 15% (12/75) of all messages were interactive messages. Finally, the program linked the interactive messages to the HMIS database of the TNHD to scan for relevant information and take the next action appropriately. For instance, if the system scans the HMIS and learns that a woman already obtained her polio shot, the system will then skip the reminder message for the polio immunization. Each type of message was sent at the appropriate time for the stage of a woman’s pregnancy; the timing was customized within the program algorithms.

### Intervention Piloting and Implementation

The intervention site was determined through discussion with the Dinh Hoa District Health Center. All communes in Dinh Hoa district were stratified into centrally located, middle and remote communes, and then random selection was applied to choose 8 project communes and 4 control communes in the district.

The mMom intervention target population included EMW living in Dinh Hoa district who had a current pregnancy or who had a child aged less than 1 year. The sampling frame within the project communes included all pregnant women, new mothers, and women of childbearing age who were known by and under the care of a village or commune health worker. All women were individually invited to participate in the project by the commune health worker in charge of her specific village. Introduction to the project and seeking informed consent for participation took place during a woman’s first prenatal visit, where a health worker described the mMom project and asked for the woman’s consent to receive SMS messages over the course of her pregnancy and participate in project evaluation activities. If she agreed, she was asked to sign a consent form and complete the preintervention survey. Her phone number and unique identity code were then added to the database, and she began to receive messages based on her estimated week of pregnancy. Once she had delivered her baby, the participant was switched to the new mother SMS program. The study was approved by an institutional review board at PHAD, which was registered with and used ethical protocols of the Office for Human Research Protections of the United States Department of Health and Human Services. Project data were subject to privacy and security protocols of the Office for Human Research Protections and the TNHD.

The mMom platform and intervention were developed over 6 months, piloted for 2 months, and implemented for 24 months. Project wrap-up occurred over the final 10 months; project data are currently under analysis, and dissemination of results is forthcoming.

### Evaluation of the Intervention’s Impact

The project evaluation framework included the following 4 major components: (1) initial ethnographic fieldwork, (2) pre- and postintervention surveys in intervention and control communes, (3) regular M&E site visits, and (4) extended mid-term and final evaluations. Each of the four components of the project evaluation framework were planned and designed at the project outset. As described earlier, ethnographic fieldwork was carried out in Dinh Hoa district at the beginning of the intervention development phase to learn about project feasibility and potential challenges and to further contextualize and refine project details. The team included experts (in obstetrics and gynecology, MNCH, public health, IT, and M&E) from Simon Fraser University (Canada), PHAD, National Hospital of Obstetrics and Gynecology (Hanoi), Hanoi Medical University, and TNHD. Focus group discussions and in-depth interviews were carried out with staff of the district and commune health centers, pregnant women and women with an infant aged less than 1 year, and family members of these target population women, especially their husbands and mothers-in-law.

Preintervention questionnaires were developed and piloted at the beginning of the project. All pregnant women and women with infants aged less than 1 year in 8 intervention and 4 control communes were invited to respond and provide information on their knowledge, attitudes, and practices around MNCH. The questionnaire was formulated to evaluate participants’ knowledge of food sources for specific nutrients, what types of experiences merited medical attention during pregnancy or new motherhood, and their attitudes toward MNCH; and to assess participants’ current mobile phone use habits, their family income, prior health care experiences, health insurance status, and other demographic details. When participants delivered their infant or experienced a miscarriage (for the pregnancy program) or when their child reached 1 year of age (for the new motherhood program) or when they wanted to cease participation for any reason, they were asked to be interviewed using a postintervention questionnaire. The postintervention questionnaire was formulated to mirror the preintervention questionnaire to assess changes in knowledge, attitudes, and practices over the course of the intervention. It is expected that this intervention-control survey design will allow us to control for confounding factors and learn about net effect of the mMom intervention with appropriate techniques, such as difference-in-differences analysis. Additionally, participants living in the 8 intervention communes were asked about their experiences with and attitudes toward the mMom project. Commune health center staff were trained to administer the questionnaires under the supervision of PHAD project officers. EpiInfo (Centers for Disease Control and Prevention, Atlanta, US) was used to develop data entry forms, and double-entry protocol was employed to control data entry quality.

Regular M&E trips were conducted by project management teams from PHAD, TNHD, and Dinh Hoa district health center to participating communes for monitoring the intervention’s progress and for anticipating and addressing any issues that arose. The mid-term and final evaluations were carried out in May 2014 and May 2016, respectively, and were led by mixed teams of semiexternal evaluators, who knew the project well but did not take part in implementing the intervention, and external evaluators (including MNCH and IT experts) to gain objective project insights. Qualitative data collection guidelines (focus group and interview guides, Multimedia Appendix 1) were developed to elicit responses from mMom stakeholders on their experiences with and perceptions of the project. Focus group discussions and in-depth interviews were carried out (by international and Vietnamese evaluators and with Vietnamese translators) with TNHD officials, district health center staff, project beneficiaries, and their families. All participants involved in evaluation activities had provided their written consent upon joining the intervention and received a small honorarium for providing their insights on the project. The focus groups and interviews were tape-recorded and transcribed. These mid-term and final evaluations provided ample qualitative data, the analysis of which is expected to support a deeper understanding of the project’s implementation and impact. Analysis of the quantitative data provided by the pre- and postintervention surveys is expected to result in quantitative evidence of the impacts of the mMom intervention on participants’ MNCH knowledge, behaviors, and interactions with the health system.

## Results

The results of the mMom project development process were as follows: (1) the successful development of the mMom system, including the mHealth platform hardware and integration into the HMIS, the intervention plan and content, project personnel, and M&E framework; (2) the piloting and implementation of the intervention through multistakeholder partnerships as planned; and (3) the implementation of all planned M&E activities.

## Discussion

The mMom project’s design and research protocol aim to contribute several lessons on multilevel stakeholder partnerships and the integration of mHealth systems with existing electronic population and health record systems, which are relatively lacking in the global mHealth literature.

The mMom system focused on development through partnerships at local, provincial, and national levels to enhance the sustainability of the intervention. One of its anticipated strengths is that the project was informed by global-level frameworks, guidance, and best practices on the application of mHealth to improve MNCH outcomes, but it was also highly context-specific because of careful attention to the local relevance of the SMS message content, a piloting phase in the Dinh Hoa district, and local implementation through existing community health work systems. The project’s high level of integration with local partners, including TNHD, the Dinh Hoa district health center, and the local commune health centers that carried out daily project activities, was anticipated to present challenges because of the significant communication necessitated by this approach. To ensure effective coordination, two dedicated project officers at PHAD communicated routinely with all partners, and the project team met the TNHD, the district health center, commune health staff, and a number of participants on at least a biannual basis to discuss the progress of the intervention and share perspectives on its future. It is expected that this consistent engagement will promote partners’ commitment to the project over the longer term.

The enabling policy and technological environment in Thai Nguyen is expected to promote this intervention’s continued integration and uptake among high-level decision makers in Dinh Hoa district and at the TNHD. As previously described, Thai Nguyen province has adopted a HMIS and has increasingly applied technology in improving health service delivery over the past two decades. In terms of technological integration, Thai Nguyen is advanced relative to other provinces in Vietnam, and officials at several health system levels have engaged with and received training in health information technologies in recent years. It was expected that this material context (ie, eHealth information components that are already in place) and a social and policy context which is committed to exploring and applying mHealth to improve health outcomes, would create an environment of high readiness for a project such as mMom and would enable the project’s effective implementation.

It was also anticipated that the mMom project would be innovative and sustainable because of its integration into the local HMIS and its implementation by the local health workers at district and commune levels. Furthermore, the TNHD was engaged in comanaging and overseeing operation of the intervention from its initiation and also took ownership of the mMom system at the official end of the project period. It was expected that continuous involvement of the health centers and government bodies at the district and provincial level would support the project’s integration into current systems and will boost the likelihood of its permanent adoption.

### Conclusions

This protocol outlines the research tools and process of the mMom project in northern Vietnam, which aims to examine the feasibility and impact of implementing an integrated mHealth intervention to improve maternal and child health knowledge and behavior among EMW living in a remote area in Vietnam. The project is likely to provide important lessons on the challenges of a highly integrated multistakeholder partnership and insights on coordination between the international project team, the provincial health department, and the district and commune health center levels, which took place as part of implementation. The project may also contribute lessons on factors that may enable or present challenges to integrating a novel component into an existing HMIS and lessons on district management of mHealth implementation. These potential contributions can provide valuable insights into currently existing gaps in the mHealth literature and are expected to support the increased integration of mHealth for MNCH projects in other contexts.

## Appendix

Multimedia Appendix 1 Qualitative data collection guidelines, mMom project evaluation.

## Abbreviations

eHealth: electronic health
EMW: ethnic minority women
HMIS: health management information system
IDRC: International Development Research Centre
IT: information technology
M&E: monitoring and evaluation
MAMA: Mobile Alliance for Maternal Action
mHealth: mobile health
MNCH: maternal, newborn and child health
PHAD: Institute of Population, Health, and Development
SMS: short message service
TNHD: Thai Nguyen Provincial Health Department
WHO: World Health Organization
