# Supplementary material for: Development of a Maternal, Newborn and Child mHealth Intervention in Thai Nguyen Province, Vietnam: Protocol for the mMom Project
Source: JMIR Res Protoc. 2018 Jan 11;7(1):e6. doi: 10.2196/resprot.7912 (PMC5785686; doi:10.2196/resprot.7912)
Supplement: Multimedia Appendix 1 [file resprot_v7i1e6_app1.pdf]

Appendix A: Qualitative data collection guidelines, mMom project evaluation

I. Focus Group Discussion – Commune health workers (Intervention)

Questions

1. What are the advantages and difficulties in implementing the project at the commune level? How are these advantages, or how are they difficulties?
2. What activities are easy to implement at the local level, and why? What activities are hard to implement, and why?
3. What problems did local project staff often have to resolve?
4. Is the content of the SMS messages easy to understand, and in accordance with the customs, traditions and language of the locals?
5. How have the mMom participants in your commune reacted to the program? How have husbands, mother and father-in laws and other family members reacted to it?
6. What is the impact of the project on health care for the local community? Can you explain this impact?
7. Has the mMom program made your work as commune health staff easier or harder? How?
8. Could the project be expanded to other communes? Why or why not?
9. If the project is implemented in other communes, what will be most difficult?
10. What are your lessons, suggestions, and recommendations for successful implementation of the project? Why do you propose such recommendations and suggestions?

II. Focus Group Discussion – Commune health workers (Control)

Questions

1. How does your commune health center currently deliver MNCH care to local women?
2. Do you face any challenges in providing care to local women? Could you describe them?
3. How do women in your commune get information about having a healthy pregnancy and delivery and keeping their infant healthy? What kind of information does the commune health centre provide?
4. What do you think are some of the barriers that women face in having healthy pregnancies and keeping their newborns healthy?
5. What are the positive systems and factors that you think help women to have healthy pregnancies and children?
6. What have you heard about the mMom program? What do you think of it?
7. Do you think the mMom program could be useful for supporting women in your commune? Why or why not?

III. Focus Group Discussion – Participant women (Intervention)

Questions

1. From what source did you learn about the mMom project? Why did you decide to participate in mMom project?
2. What are the advantages and disadvantages that you have experienced since you joined the project? Can you explain these?
3. When you have a problem with your health or your child’s health, do you feel confident to call the commune health centre? Why or why not? Have the commune health staff promptly resolved your problem? Why or why not?
4. Which mMom messages did you not understand clearly? Why?
5. When you do not understand a message, do you ask the commune health staff? Why or why not?
6. Have you ever received explanations of the messages from the health staff? Were you satisfied with these explanations?
7. Have your husband and family members supported your involvement in the project? In what ways have they supported you or not supported you?

8. Do you think the messages you received helped you take better care of your health and your children's health? How did they help?
9. Are you satisfied with the message service of the mMom project? Why?
10. If you had to pay for the service to get messages about health care for mothers and children, would you be willing to pay? How much? Why or why not?
11. Do you have any suggestions for this project to make it better in the future?

IV. Focus Group Discussion – Participant women (Control)

Questions

1. From what sources have you ever received information about health care for mothers and children?
2. How do you use this information?
3. When you have a problem with your health or your child's health, do you feel confident to call the commune health centre? Why or why not? Have the health staff promptly resolved your problem? Why or why not?
4. Have you heard about the mMom project? If so, from what source?
5. Do you think receiving SMS messages about health care is useful for mothers in taking care of their heath and their children's health? Why?
6. Would you want to join the project? Why?
7. If you had to pay for the service to get messages about health care for mothers and children, would you be willing to pay? How much? Why or why not?

V. In-depth interviews – Thai Nguyen Provincial Health Department

Questions

1. How does TNHD think about the mMom project?
2. What is the impact of the project on health care for the local community? Can you explain this impact?
3. From the perspective of TNHD, what are the advantages and difficulties in implementing the project at the provincial levels? How are these advantages, or how are they difficulties? (they might relate to management, system operation, work with the commune level)
4. What are your suggestions, and recommendations for successful implementation of the project? Why do you propose such recommendations and suggestions?
5. Do you believe the mMom project could be sustained in the current communes?
6. If the project could be scaled up to reach more communes throughout the province, which would be prepared and which factors should be improved?

VI. In-depth interviews – Dinh Hoa district health centre

Questions

1. How does DHDHC think about the mMom project?
2. What is the impact of the project on health care for the women involved? What about the impact on their children and their family life? Can you explain this impact?
3. Do you think that the project has helped to address barriers faced by women in seeking MNCH care? If so, how?
4. From the perspective of DHDHC, what are the advantages and difficulties in implementing the project at the district level? How are these advantages, or how are they difficulties?
5. What are your lessons, suggestions, and recommendations for successful implementation of the project? Why do you propose such recommendations and suggestions?
6. Do you believe the mMom project could be sustained in the current communes?
7. If the project could be scaled up to reach more communes throughout the district? Which factors should be improved?
8. How would DHDHC be willing to support future efforts to implement the project?

VII. In-depth interviews – Commune Health Workers (Intervention)

Questions

1. What work are you assigned to do in the project? Does your role suit your abilities? Why or why not?
2. In addition to your assigned role in the project, is there other work related to the project that you have had to do recently? If so, what is the work specifically?
3. According to you, has the project achieved good results after 2 years of implementation? Why do you think it has or hasn't?
4. What are the advantages and disadvantages of implementing the mMom project in your community? Why are these advantages, or disadvantages?
5. Do you think that the women participants are satisfied with the project? If so, how are they satisfied? Can you give a few examples? If not, do you know why they are not satisfied with the project?
6. What are the impacts of the project on women and children's health in the commune?
7. Is the content of the SMS messages easy to understand, in accordance with the customs, traditions and language of the local community?
8. Do you feel that mMom project participants have been able to take advantage of the program and maximize the information from the messages? If not, what do you think the barriers are?
9. According to you, what is necessary for this project to be maintained sustainably in the local commune? Why?
10. According to you, could this project be extended to other communes? If so, what work has to be done? If not, why not?
11. What are your lessons, proposals, or suggestions for successful implementation of the project objectives? Can you explain why?

VIII. In-depth interviews – Commune Health Workers (Control)

Questions:

1. Are you mainly responsible for implementing the health care of mothers and children in the commune? Please describe your work. Is this work appropriate for you? Why?
2. Have you ever actively reached out to women of childbearing ages in the commune? If yes, how did you approach them? Why did you do so? If not, why?
3. Have you ever been directly involved in educational or promotional communication encouraging women to think about their own or their children's health? If so, please describe what you were doing. Do you think that way is effective?
4. If you do not directly perform any of the above activities, who from the commune health centre did so? Do you know how did they do it?
5. How much have you heard about the mMom project?
6. Do you think that you would have the capacity to implement the project in your commune? Why or why not?
7. If the project was implemented in your commune, do you think it would succeed? Why or why not?
8. How do you think women in your commune would react to the mMom project?

IX. In-depth interviews – Participant women (Intervention)

Questions

1. From what source did you learn about the mMom project?
2. What are the advantages and disadvantages that you have experienced since you joined the project? Can you explain these?
3. When you have a problem with your health or your child's health, do you feel confident to call the commune health centre? Why or why not? Have the health staff promptly resolved your problem? Why or why not?

4. Which mMom messages did you not understand clearly? Why?
5. When you do not understand a message, do you ask the health staff? Why or why not?
6. Have you ever received explanations of the messages from the health staff? Were you satisfied with these explanations?
7. Have you talked with your husband and family members about message service of the mMom project?
8. Have your husband and family members supported your involvement in the project? In what ways have they supported you or not supported you?
9. Do you think the messages you received helped you take better care of your health and your children's health? How did they help?
10. Are you satisfied with the message service of the mMom project? Why?
11. If you had to pay for the service to get messages about health care for mothers and children, would you be willing to pay? How much? Why or why not?
12. Do you have any suggestions for this project to make it better in the future?

X. In-depth interviews - Family members of participant women (Intervention)

Questions

1. How do women and children in your community usually get health care?
2. Where do you learn about mMom project?
3. Which of your family members is involved in the mMom project?
4. What are the advantages and disadvantages that you think she has experienced since joining the project? Can you explain these?
5. Do you support her being involved in the project and receiving the messages? Why, or why not?
6. Do you think the messages she received helped her take better care of her health and the children's health? How did they help?
7. Do want to participate in mMom project and receive SMS to take care your wife and your baby better? Why or why not?
8. If the mMom project was going to be implemented again in the community, what changes do you think should be made to the project or the messages? Why?

XI. In-depth interviews – Participant women (Control)

Questions

1. From what sources have you ever received information about health care for mothers and children?
2. How do you use this information?
3. When you have a problem with your health or your child's health, do you feel confident to call the commune health centre? Why or why not? Have the health staff promptly resolved your problem? Why or why not?
4. Have you heard about the mMom project? If so, from what source?
5. Do you think receiving SMS messages about health care is useful for mothers in taking care of their heath and their children's health? Why?
6. Would you want to join the project? Why?
7. If you had to pay for the service to get messages about health care for mothers and children, would you be willing to pay? How much? Why or why not?
